# Perception of the multidisciplinary team and the patient's family companion regarding their presence during emergency room care

**DOI:** 10.31744/einstein_journal/2025AO0871

**Published:** 2025-06-10

**Authors:** Juanna Elisa Oliveira, Rafael da Silva Giannasi Severini, Gabriella Trevisan Padilha, Amélia Gorete Afonso da Costa Reis, Sylvia Costa Lima Farhat, Cláudio Schvartsman

**Affiliations:** 1 Universidade de São Paulo Faculdade de Medicina Hospital das Clínicas São Paulo SP Brazil Instituto da Criança, Hospital das Clínicas, Faculdade de Medicina, Universidade de São Paulo, São Paulo, SP, Brazil.

**Keywords:** Child, Adolescent, Cardiopulmonary resuscitation, Intubation, Intubation, intratracheal, Patient care team, Emergency service, hospital, Hospital, pediatric, Surveys and questionnaires

## Abstract

**Objective:**

To evaluate the perceptions of family companions and the professional team regarding the presence of a family member during emergency and urgent care at a tertiary pediatric hospital.

**Methods:**

This exploratory, descriptive cross-sectional study was conducted at a tertiary pediatric hospital that mandated the presence of companions in the emergency room during emergency care and/or procedures. The researcher conducted interviews after emergency care and administered questionnaires to the participants.

**Results:**

Ninety-five emergency room visits were selected, resulting in 92 interviews with companions and 148 interviews with multidisciplinary team members. Of the companions, 71% were unaware of the legislation regarding their presence. Despite their lack of knowledge, 90.2% insisted on accompanying the patient. Of the multidisciplinary team members, 37% were unaware that the patient could be accompanied to the emergency room, and 2% reported not allowing a companion in the emergency room. Nevertheless, 87% of the team agreed that the companion comforted the patient during care in the emergency room.

**Conclusion:**

Results from both companions and health professionals perceive the companion's presence during emergency room care as positive, making it necessary to implement care protocols that supports a companion's presence during pediatric care.

## In Brief

In this cross-sectional study, we explored the perceptions of family companions and multidisciplinary teams regarding family presence during pediatric emergency care. This study's findings revealed positive effects on patient comfort and communication, highlighting the need for well-defined policies and team training.

## Highlights

■ Family presence improves communication and patient comfort during pediatric emergency room care.
■ Overall, 90.2% of companions stayed during care, despite 71% being unaware of the legislation.
■ Of the professionals, 96% allowed companions, with 87% citing comfort for patients.
■ This study supports creating clear protocols for family inclusion during emergencies.

## Graphical abstract

**Figure:**
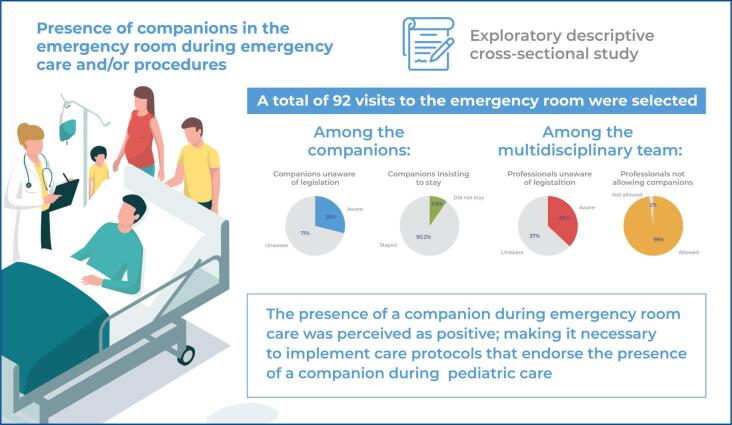

## INTRODUCTION

The Brazilian health system was established by the Brazilian Federal Constitution of 1988,^(1)^ and has since been known as the Unified Health System (SUS - *Sistema Único de Saúde*). In this study, we aimed to provide universal, comprehensive, and free public health system access without discrimination. Since its creation, the health system has been categorized into three levels of care: Primary, through Basic Health Units (UBS - *Unidades Básicas de Saúde*) serving as the patient's first step when using the system; secondary, through specialized services offered by hospitals and outpatient clinics providing care organized by medical specialties; and tertiary, encompassing more highly complex care in advanced hospitals that generally require cutting-edge technology.^(2)^

*Instituto da Criança e do Adolescente do Hospital das Clínicas da Faculdade de Medicina da Universidade de São Paulo* (FMUSP), a tertiary pediatric care service, became the National Reference Center for Child and Adolescent Health in 1999, following an agreement between the FMUSP's Department of Pediatrics, Ministry of Health, and the São Paulo Municipal Health Department. This study, conducted at this institution, is part of a master's dissertation^(3)^ investigating the perception of companions and healthcare professionals regarding family member's presence during emergency care.

Article 12 of the Brazilian Child and Adolescent Statute^(4)^ states that "Health care establishments must provide necessary conditions for the full-time stay of one of the parents or guardians, in cases of hospitalization of a child or adolescent" (loosely translated). However, no specificity regarding the companion's presence during emergency room care was established.

Acknowledging the particularities of emergency care, the 2009 Brazilian National Humanization Policy (PNH - *Política Nacional de Humanização*),^(5)^ proposed changes to how the system is managed and the care patients receive in 2013. Since then, new possibilities for healthcare have been presented, considering a healthcare system focused on the individual, including patients, managers, and workers. One of these proposals was the presence of a family companion to improve patients’ experiences during complex emergency care.

Brazilian Law No. 14.364,^(6)^ enacted on January 6, 2022, guarantees the rights of companions of all patients with priority care. However, nothing specific was added regarding a companion's presence during emergency room care.

Despite this, the presence of companion during emergency room visits and invasive procedures is encouraged for pediatric patients. International associations, such as the American Heart Association and the Emergency Nurses Association, recommend family presence in the emergency room during invasive procedures and cardiopulmonary resuscitation (CPR).^(7,8)^ However, in Brazil, parental participation in these services remains infrequent, as observed by Reis.^(9)^

## OBJECTIVE

To evaluate the perceptions of family companions who witnessed emergency care and healthcare professionals providing emergency care in their presence.

## METHODS

In this exploratory, descriptive, cross-sectional study, we collected data through researcher-administered questionnaires during post-emergency care interviews. following signing of informed consent forms.

The study was conducted in the Emergency Room of the *Instituto da Criança e do Adolescente*, part of the *Hospital das Clínicas*, attached to the *Faculdade de Medicina da USP* (HCFMUSP), where it is the norm to offer companions the option of staying in the emergency room during care and/or for procedures.

The researcher administered questionnaires to companions and the health professionals from the multidisciplinary team involved in the emergency care. Satisfaction was assessed using a Likert scale of 1-5 (1 = strongly disagree >5 = strongly agree).^(10)^ To mitigate all response bias, key questions were adopted, with half of the questionnaires applied using the affirmative option and the other half, negative, such as: "Are you satisfied with having been present in the emergency room?" and "Are you dissatisfied with having been present in the emergency room?".

Data were collected through interviews conducted between August 2019 and November 2021. Questionnaires covered demographic data and perceptions of companions (Table 1S, Supplementary Material) regarding their stay in the emergency room and health professionals (Table 2S, Supplementary Material) regarding the presence of a companion in the emergency room.

Between August 2019 and November 2021, 24,127 patients were seen in the Emergency Room, with 1,117 classified as orange/red, which, according to the severity scale used by the service, indicates immediate care in the emergency room.

Ninety-five cases met the following inclusion criteria: cardiorespiratory arrest, orotracheal intubation, and/or the need for intraosseous and/or central access and/or hemodynamic instability. These criteria resulted in 92 interviews with companions and 148 with the multidisciplinary team. Finally, three cases were excluded due to an unsigned informed consent form.

### Statistical analysis

Continuous parametric variables are presented as mean and standard deviation, and non-parametric variables as median and range (minimum-maximum).

Categorical variables are presented as frequencies and percentages. Satisfaction survey results included mean ± standard deviation, or median and variation, and the percentages for each Likert scale level, divided by statement. A significance level of 5% was used for all the analyses. All data were analyzed using the Statistical Package for Social Sciences 22.0 software.

The study was approved by the HCFMUSP Ethics and Research Committee (CEP), CAAE: 93344418.1.0000.0068; # 3.009.260, and all participants signed an informed consent form before the interviews.

## RESULTS

In total, 92 interviews were conducted with companions, and 148 were conducted with a multidisciplinary team. Interviews with companions: Mothers of the children and adolescents comprised the majority of companions (88%); of those, 43% were the primary income earners in the family and received assistance benefits or alimony, while the other 57% were heads of their families and received assistance benefits. In addition, most cases (57%) with mothers as companions belonged to a traditional nuclear family (fathers, mothers, and children).

The most prevalent age group of companions was 18–30 years (46.7%), and only 4.3% were >50 years old.

Regarding educational background, 63% had completed high school, and only 1.1% were illiterate.

Notably, most patients receiving emergency room care included in this study had some chronic disease, with an average period of 3.13 (±3.4) years of outpatient follow-up at our hospital.

The most prevalent diseases were neurological in 36 cases (33.1%), gastrointestinal/hepatic in 15 (14%), respiratory in 13 (12%), oncological/hematological in 12 (11%), genetic in 7 (6%), renal in 3 (3%); with 5 having other ailment (5%).

In 52.7% of the interviews, companions stated that the patient had prior emergency care.

The majority (71.0%) of companions were unaware that they could remain with the patient during emergency room care. However, 90.2% insisted on staying with the patient during emergency room care, and 56.5% had witnessed a prior form of emergency care for the patient.

The average time from the worsening of the disease to arrival at the emergency room was 1.95 (±2.1) days.

Regarding the procedures, 88% of the patients required venous access due to hemodynamic instability, 31.5% required oral tracheal intubation, and 4.3% required cardiopulmonary resuscitation. Only 5% of the patients died.

Companions indicated that the team communicated with them during emergency care in 97.9% of the cases (4 and 5 on the scale), explained the severity of the case during the emergency care process in 90.2% of the cases, and completely agreed that they received adequate support from the team in 96.7% of the cases (Table 1).

**Table 1:** Companions’ perception regarding their presence during emergency care of the patient for whom they were responsible

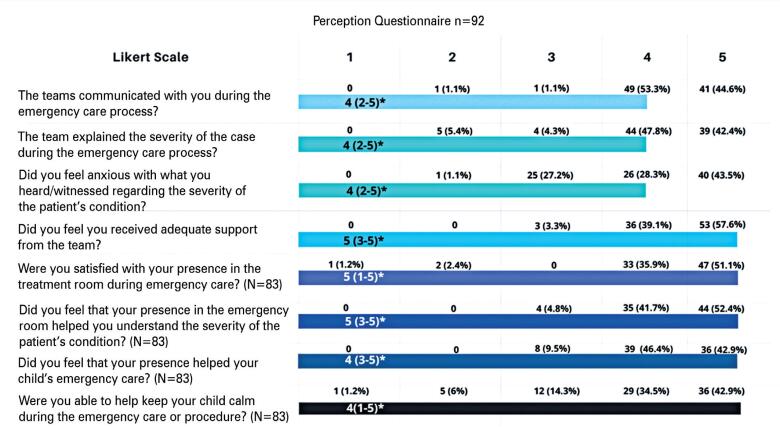

* median and range.

Eighty-seven percent of companions reported satisfaction with their presence, whereas 94.1% felt that their presence during emergency care of the patient for whom they were responsible was crucial for them to understand the case severity.

Regarding interviews with the multidisciplinary team, 147 health professionals were interviewed: 69% doctors, 22% nurses, and 9% physiotherapists.

Of all the healthcare professionals interviewed, 85% were aged ≤40 years, 81% were women, and 80% had <10 years of experience.

Notably, 96% of the interviewees allowed companions to remain in the emergency room during care, although only 63% reported knowing about the hospital's policy or legislation beforehand. Of the 55 health professionals unaware of the policy or legislation, 98% (54) allowed companions during emergency care. Of the 96% who allowed companions, 15% disagreed with the policy/legislation or were indifferent to the presence of companions during emergency care. Of those who allowed companions, 55% were 30 or younger, 77% had <10 years of experience, and 41% were doctors.

Only four health professionals barred their companions from the room: two physiotherapists, one resident doctor, and one nursing assistant. Three knew the policy/legislation and had less than 10 years of experience.

Only 8% of respondents felt uncomfortable with companion presence in the emergency room, while 92% felt comfortable. Of those interviewed, 87% did not think that companion presence during emergency care hindered case management, whereas 87% agreed that companion presence provided patient comfort.

In adddition, 93% of those interviewed agreed that companions could better understand the severity of the case by staying with them during emergency care.

In 88% of the cases, one healthcare professional was responsible for talking/interacting with the companion during care.

## DISCUSSION

In this study, we aimed to describe the perceptions of companions and the multidisciplinary team regarding companion presence during emergency/urgent care in a tertiary pediatric hospital.

The data collected revealed that most companions (88%) were mothers of the children and adolescents under care, with 43% being the family's primary income earners receiving assistance benefits or alimony, facilitating their presence in the hospital due to lack of formal employment.

While Brazilian society has historically consisted of nuclear families with husbands, wives, and children, recent trends indicate an increase in single-parent families over the last few years. However, this study observed a higher prevalence of nuclear families among mother-accompanied patients in the emergency room.

Mothers who are the family's primary earners often face significant challenges, needing to juggle multiple responsibilities, including financial responsibilities, while still caring for children, managing the home, and dealing with emotional issues resulting in additional pressure and stress. An aggravating factor in the context of this study was that most patients required additional care. Regarding social assistance, it is important to assess families’ specific needs to ensure access to health services, education, housing, and financial support.

In this study, 96% of companions were unaware of their right to remain with the patient during emergency room care, whereas 63% of healthcare professionals reported having this knowledge. Despite this low number of health professionals, 98% allowed companions presence in the emergency room.

Companions educational background showed that 64.1% had completed only secondary education, 23.9% only basic education, and 1.1% were illiterate. This low educational level translated into an almost general lack of interest in seeking legislation and an equally low interest in public policies to make this legislation known to all.

A systematic review by Boudreaux et al.^(11)^ identified 30 articles that studied the companion's presence during invasive procedures and cardiopulmonary resuscitation. Of these, 20 were selected for further review and divided into two groups: the first regarding companion presence during invasive procedures and resuscitation. In the first group, 62% of parents remained with their child during the emergency procedure, and 78% demonstrated interest in remaining with them during invasive procedures. In the second group, most family members wanted to be present again, if necessary. Using Fisher's exact test and content analysis, 80% of the family members wanted to be present during procedures. Despite separating them into two groups, the study concluded that companions with previous experience generally favored being present during invasive procedures in the emergency room.

McGahey-Oakland et al.^(12)^ conducted a descriptive and retrospective study in 2007 analyzing the experiences of 10 companions during CPR. They concluded that even when companions opted not to be present during CPR, it was important to offer this option, regardless of formal policies. In the tertiary hospital where this study was conducted, it is common practice for companions to be offered this option, which was identified by 90.2% of the companions who remained in the emergency room during care.

A descriptive study conducted by Mangurten et al.^(13)^ evaluated the effects of family presence during resuscitation and invasive procedures in a pediatric emergency unit. All 22 interviewees emphasized the importance of a companion's presence during emergency care. Most believed they had the right to be present during invasive procedures, even if their presence did not change how patients received care. Similarly, in our study, approximately 90% of companions remained with the patient during emergency care. The companions perceived their presence positively, citing quick response time and satisfaction in staying together during the entire procedure, despite fear of bad outcomes being the most cited feeling among them. They demonstrated a satisfactory level of trust in the multidisciplinary health team, as their presence allowed them to see the entire procedure and better understand everything that was happening, including the effort made by the team to provide the best care possible.

A European systematic review of 36 articles on family presence during pediatric resuscitation conducted by Dainty et al.^(14)^ found that most companions wanted the option to be present during emergency care, particularly in situations where IP or CPR was required. The review also found that 40% of companions were not present in the emergency room because they were not offered this option. Of those who were not present, 55% expressed interest in the possibility of being present and tended to recommend to other families that they be present during resuscitation if they had the option.

Most companions in the present study reported positive communication with them during care and that they had received adequate support. In 88% of the cases, a healthcare professional was responsible for talking to the companion during care.

Effective, clear, and objective communication is crucial for the multidisciplinary team to engage patient's family, ensuring that the family member can contribute to the care and that the team's efforts are made evident to everyone involved, thus easing the mourning process if it occurs. This stance is defended by Sucupira et al.,^(15)^ highlighting the importance of verbal and nonverbal communication during care in improving the quality of care provided, thus welcoming companions in a humanized way.

Our study's multidisciplinary team was predominantly women, with 41% of the health professionals being doctors and 85% aged 40 years or less. According to Technical Report no. 4/2021^(16)^ the ProvMed 2030 – a study that supports the National Plan for Strengthening Health Residencies of the Ministry of Health, carried out in partnership between the Ministry of Health and the USP – projects that, in seven years, most doctors will be women, 80% between the ages of 22 and 45 years.

In a systematic review by Boudreaux et al.^(11)^ the presence of companions during CPR was greater when performed by nurses (96%) than by emergency room physicians (79%). In our study, 96% of the multidisciplinary team interviewed allowed companion presence during care, with only 15% disagreeing with or being indifferent to their presence in the emergency room.

A descriptive study by Mangurten et al.^(13)^ found that 94% of the interviewed professionals felt comfortable with their presence in the emergency room, with 89% reporting no change in performance during the procedure. These findings align with our study, where 96% of those interviewed by the multidisciplinary team stated that they allowed companions to remain during emergency care, and only 8% felt uncomfortable with their presence during care. When health professionals were asked whether they agreed with their companions’ presence in the emergency room, 85% said yes. Of these, 55% were 30 or younger, 77% had <10 years of experience, and 41% were doctors. This result is consistent with the cross-sectional study by Mekitarian et al.,^(17)^ who concluded that less experienced professionals (<10 years) were more likely to favor family members’ presence during tracheal intubation and CPR procedures. In comparison, this percentage decreased among those with more than 10 years of experience. Unlike the study by Mekitarian et al., our results demonstrate that practically all professionals, regardless of their years of experience, allowed companions to be present during emergency room care.

In our study, 75.5% of the multidisciplinary team felt comfortable with companion presence during emergency care, while 87% of the health professionals interviewed did not believe that companion presence hindered case management. They also recognized its comfort to the patient during emergency care and its role in improving family members’ understanding of the case severity. These results contradict the cross-sectional study by Mekitarian et al.^(17)^ carried out with 46 health professionals (medical and nursing teams), which concluded that the more invasive the procedure, the fewer professionals favored companions. The study also reported reasons why the multidisciplinary team usually chose to include a companion during emergency room care included the following: the family witnessing life-saving efforts, the family providing important information about the patient, because it was the family's right, and because the family provided comfort to the child, all similar to the data collected in our study. Mekitarian et al.^(17)^ also noted that a companion's presence during emergency care tends to help the grieving process if death occurs, making communication more effective and allowing companions to witness all efforts made to save the patient's life. Our study corroborates these results, with 93% of the professionals agreeing that companion presence during emergency care improves family understanding of the case severity.

Allowing companions to be present during emergency care is a discussion that continues since companions can contribute during care. This study identified the need to improve training, communication skills, and awareness of multidisciplinary teams through well-defined protocols.

## CONCLUSION

The presence of a companion during invasive procedures in the emergency room is perceived as positive by the companion and the multidisciplinary team. Despite not being widespread in Brazil, will likely increase as medicine is increasingly centered on family and patients.

It is imperative that during emergency room care, one professional is responsible for mediating communication between the team and the companion, allowing the team to work better and without pressure. In this study, we highlighted the need to develop specific protocols to ensure that companions are present or absent during emergency care to train multidisciplinary teams.

## Perception of the multidisciplinary team and the patient's family companion regarding their presence during emergency room care

Juanna Elisa Oliveira, Rafael da Silva Giannasi Severini, Gabriella Trevisan Padilha, Amélia Gorete Afonso da Costa Reis, Sylvia Costa Lima Farhat, Cláudio Schvartsman

**DOI: 10.31744/einstein_journal/2025AO0871**

### Table 1S Questionnaire for the patients companions in emergency care

**Table:**

**Positive Questionnaire for the Emergency Patient's Companion**
Companion: ( ) Father ( ) Mother ( ) Legal Guardian ( ) Other
Age of the Companion: ( ) 18 – 30 years ( ) 31 – 40 years ( ) 41 – 50 years ( ) 51 or more years
Education Level: ( ) Illiterate ( ) Elementary School ( ) High School ( ) Higher Education
Profession:
1. Do you know the legislation regarding staying with the patient? ( ) Yes ( ) No
2. Did you want to stay with the patient during emergency care? ( ) Yes ( ) No Why?
3. Did the team communicate with you during the emergency care process? 1 - Strongly Disagree 2 - Disagree 3 - Neutral 4 - Agree 5 - Strongly Agree
4. Did the team explain the severity of the case to you during the emergency care process? 1 - Strongly Disagree 2 - Disagree 3 - Neutral 4 - Agree 5 - Strongly Agree
5. Did you feel anxious about what you heard/saw regarding the patient's condition? 1 - Strongly Disagree 2 - Disagree 3 - Neutral 4 - Agree 5 - Strongly Agree
6. Did you feel that you received adequate support from the team (e.g., doctors, nurses, social workers) during and after emergency care? 1 - Strongly Disagree 2 - Disagree 3 - Neutral 4 - Agree 5 - Strongly Agree
7. Were you satisfied with staying in the emergency room during the emergency? ( ) Not applicable 1 - Strongly Disagree 2 - Disagree 3 - Neutral 4 - Agree 5 - Strongly Agree
8. Do you feel that your presence in the emergency room helped you understand the severity of the case? ( ) Not applicable 1 - Strongly Disagree 2 - Disagree 3 - Neutral 4 - Agree 5 - Strongly Agree Please explain:
9. Do you think your presence helped in your child's emergency care? ( ) Not applicable 1 - Strongly Disagree 2 - Disagree 3 - Neutral 4 - Agree 5 - Strongly Agree
10. Were you able to calm your child during the emergency care/procedure? ( ) Not applicable 1 - Strongly Disagree 2 - Disagree 3 - Neutral 4 - Agree 5 - Strongly Agree
11. How do you feel about being present or not in the emergency room?
**Negative Questionnaire for the Emergency Patient's Companion**
Companion: ( ) Father ( ) Mother ( ) Legal Guardian ( ) Other
Age of the Companion: ( ) 18 - 30 years ( ) 31 – 40 years ( ) 41 – 50 years ( ) 51 or more years
Education Level: ( ) Illiterate ( ) Elementary School ( ) High School ( ) Higher Education
Profession:
12. Do you know the legislation regarding staying with the patient? ( ) Yes ( ) No
13. Did you want to stay with the patient during emergency care? ( ) Yes ( ) No Why?
14. Did the team communicate with you during the emergency care process? 1 - Strongly Disagree 2 - Disagree 3 - Neutral 4 - Agree 5 - Strongly Agree
15. Did the team explain the severity of the case to you during the emergency care process? 1 - Strongly Disagree 2 - Disagree 3 - Neutral 4 - Agree 5 - Strongly Agree
16. Did you feel anxious about what you heard/saw regarding the patient's condition? 1 - Strongly Disagree 2 - Disagree 3 - Neutral 4 - Agree 5 - Strongly Agree
17. Did you feel that you received adequate support from the team (e.g., doctors, nurses, social workers) during and after emergency care? 1 - Strongly Disagree 2 - Disagree 3 - Neutral 4 - Agree 5 - Strongly Agree
18. Were you satisfied with not staying in the emergency room during the emergency? ( ) Not applicable 1 - Strongly Disagree 2 - Disagree 3 - Neutral 4 - Agree 5 - Strongly Agree
19. Do you feel that your presence in the emergency room helped you understand the severity of the case? ( ) Not applicable 1 - Strongly Disagree 2 - Disagree 3 - Neutral 4 - Agree 5 - Strongly Agree Please explain:
20. Do you think your presence helped in your child's emergency care? ( ) Not applicable 1 - Strongly Disagree 2 - Disagree 3 - Neutral 4 - Agree 5 - Strongly Agree
21. Were you able to calm your child during the emergency care/procedure? ( ) Not applicable 1 - Strongly Disagree 2 - Disagree 3 - Neutral 4 - Agree 5 - Strongly Agree
22. How do you feel about being present or not in the emergency room?

### Table 2S Questionnaire for the Emergency Care Professional

**Table:**

Professional Role: ( ) Attending Physician ( ) Resident Physician ( ) Preceptor Physician ( ) Nursing Assistant ( ) Nurse
Age of the Professional: ( ) 18 - 30 years ( ) 31 – 40 years ( ) 41 – 50 years ( ) 51 or more years
Years Since Graduation: ( ) <10 years ( ) 10–20 years ( ) 21–30 years ( ) 31 or more years
1. Do you know the legislation regarding patient accompaniment? ( ) Yes ( ) No
2. Do you think family members should be present in the emergency room during care? 1 - Strongly Disagree 2 - Disagree 3 - Neutral 4 - Agree 5 - Strongly Agree Why?
3. Have you ever allowed a family member to be present in the emergency room during care? ( ) Yes ( ) No If yes, how many times?
4. In which emergency procedures do you consider it appropriate for family members to be present?
5. Was someone on the team responsible for talking to the companion during the emergency care process? ( ) Yes ( ) No
6. Did you feel uncomfortable having a family member present in the emergency room? 1 - Strongly Disagree 2 - Disagree 3 - Neutral 4 - Agree 5 - Strongly Agree
7. Do you feel that the presence of a family member in the emergency room made procedures more difficult? 1 - Strongly Disagree 2 - Disagree 3 - Neutral 4 - Agree 5 - Strongly Agree
8. Do you think the family member provided comfort to the patient during and/or after the emergency procedure? 1 - Strongly Disagree 2 - Disagree 3 - Neutral 4 - Agree 5 - Strongly Agree
9. Do you think the family member understood the severity of the case better by witnessing the emergency care? 1 - Strongly Disagree 2 - Disagree 3 - Neutral 4 - Agree 5 - Strongly Agree
